# Surface Modification of Poly(l-lactic acid) through Stereocomplexation with Enantiomeric Poly(d-lactic acid) and Its Copolymer

**DOI:** 10.3390/polym13111757

**Published:** 2021-05-27

**Authors:** Qianjin Zhu, Kaixin Chang, Liyan Qi, Xinyi Li, Woming Gao, Qinwei Gao

**Affiliations:** 1 College of Chemical Engineering, Nanjing Forestry University, Nanjing 210037, China; 18260077835@163.com (Q.Z.); ckx851127@gmail.com (K.C.); 18362985223@163.com (L.Q.); xinyilee7@163.com (X.L.); orangtreechina@gmail.com (W.G.); 2 Co-Innovation Center of Efficient Processing and Utilization of Forest Resources, Nanjing Forestry University, Nanjing 210037, China

**Keywords:** poly(l-lactic acid), poly(d-lactic acid), poly(d-lactic acid-*co*-glucose), stereocomplex, surface modification

## Abstract

Poly(l-lactic acid) with high molecular weight was used to prepare PLLA films by means of the solvent casting technique. Poly(d-lactic acid) (PDLA) and poly(d-lactic acid-*co*-glucose) copolymer (PDLAG) with a low molecular weight were synthesized from d-lactic acid and glucose through melt polycondensation. PLLA films were immersed in PDLA or PDLAG solution to prepare surface-modified PLLA films. The modified PLLA film presented stereocomplex crystal (SC) on its surface and homogeneous crystals (HC) in its bulk. The HC structure and surface morphology of modified PLLA films were obviously damaged by PDLA or PDLAG solution. With increasing immersion time, the PLLA films modified by PDLA decreased both the HC and SC structure, while the PLLA films modified by PDLAG increased the SC structure and decreased the HC structure. Hydrophilic glucose residues of PDLAG on the surface would improve the hydrophilicity of surface-modified PLLA films. Moreover, the hydrophilicity of glucose residues and the interaction of glucose residues with lactic acid units could retard HC destruction and SC crystallization, so that PLLA films modified by PDLAG possessed lower melting temperatures of HC and SC, the crystallinity of SC and the water contact angle, compared with PDLAG-modified PLLA films. The SC structure could improve the heat resistance of modified PLLA film, but glucose residues could block crystallization to promote the thermal degradation of PLA materials. The surface modification of PLLA films will improve the thermal stability, hydrophilicity and crystallization properties of PLA materials, which is essential in order to obtain PLA-based biomaterials.

## 1. Introduction

Poly(lactic acid) (PLA) can replace traditional plastic in food packaging [1,2,3,4,5,6], bioengineering materials [7,8,9], composites [10,11,12] and other fields due to its good biodegradability, biocompatibility, and processability. However, PLA still has many inherent defects, such as the long degradation period and poor hydrophilicity, as well as low heat resistance which may increase PLA processing difficulty [13,14,15,16].

The blending modification of PLA is one of the important ways to improve PLA performance, which will extend the application of PLA materials [17,18,19]. In 1987, Ikada [20] first blended poly(l-lactic acid) (PLLA) and poly(d-lactic acid) (PDLA) in solutions to obtain the stereocomplex of PLA (sc-PLA) with a melting point about 50 °C higher than that of PLLA or PDLA, which can effectively improve PLA heat resistance, thereby reducing PLA processing difficulty and expanding PLA application fields. Enantiomeric PDLA and PLLA chains in close contact can form stereocomplex crystal (SC) in sc-PLA through hydrogen bonding. Ajiro [21] controlled the mobility of PDLA and PLLA chains to conduct stereocomplex crystallization owing to the good chain mobility of PDLA and PLLA chains. Tretinnikov [22] found that PDLA could be selectively adsorbed on the surface of PLLA via stereocomplexation between PLLA and PDLA. 

Many researchers have recently devoted attention to the surface modification of PLA in order to improve PLA performance and expand its applications [4,23,24,25,26,27,28]. Aulin [29] built up a transparent nano-cellulose multilayer film on PLA with adjustable gas barrier properties using the layer-by-layer deposition method. Albertsson [30] utilized the “grafting” method to obtain an electroactive hydrophilic PLA surface through covalent modification with tetraaniline.

In our previous papers, we prepared poly(l-lactic acid-*co*-glucose) copolymer (PLLAG) and poly(d-lactic acid-*co*-glucose) copolymer (PDLAG) from l-lactic acid (l-LA), d-lactic acid (d-LA) and glucose, respectively, through melt copolymerization. The average molecular weights (*M*_w_) of PLLAG and PDLAG were about 15,600–20,600 with the PDI being about 1.90. PLLAG and PDLAG were blended to obtain sugar-containing stereocomplexes of PLA (sc-PLAG) [31,32], which resulted in higher heat-resistance and better hydrophilicity of sc-PLAG compared with PLLAG and PDLAG. Due to low *M*_w_ usually reducing PLA properties, in this paper, we utilized a PLLA with high molecular weight (*M*_w_) to prepare PLLA film, and surface-modified the film with low-*M*_w_ PDLA and PDLAG through stereocomplexation of enantiomeric PLA chains with amphiphilicity due to glucose residues and lactic acid segments. Low-*M*_w_ PDLA and PDLAG were synthesized from d-LA and glucose through melt copolycondensation. PLLA films were prepared by the solvent casting technique, and immersed in PDLA or PDLAG solution to proceed with surface-modification. The stereocomplex of PLLA with PDLA or PDLAG would form on the surface of modified PLLA films, while glucose residues of PDLAG sticking to the surface would improve the hydrophilicity of these films. Stereocomplex formation between enantiomeric sections of PLLA with PDLA or PDLAG is related to the increase in the *T*_m_ of PLLA [31,32]. Therefore, the surface modification of PLLA films would improve the thermostability and hydrophilicity, and change the surface structure of PLA materials, which is essential in order to obtain PLA-based biomaterials. The surface modified PLA films can be used in food packaging and biological materials.

## 2. Materials and Methods

### 2.1. Materials

Poly(l-lactic acid) (Ningbo Global Biomaterials Co., Ltd., Ningbo, China), d-lactic acid(A.R., 90%, Musashino Chemical Co., Ltd., Yichun, China), Anhydrous glucose(A.R., Sinopharm Chemical Reagent Co., Ltd., Shanghai, China), Stannous chloride(A.R., Shanghai Jiuyi Chemical Reagent Company, Shanghai, China), p-Toluenesulfonic acid(A.R., Shanghai Lingfeng Chemical Reagent Company, Shanghai, China), Stannous octoate(A.R., Sinopharm Chemical Reagent Co., Ltd., Shanghai, China), Methanol(A.R., Nanjing Chemical Reagent Co., Ltd., Nanjing, China), Trichloromethane (A.R., Shanghai Pilot Chemical Corporation, Shanghai, China), other reagents are of analytical grade.

### 2.2. Preparation of Samples

#### 2.2.1. Synthesis of Poly(d-lactic acid) (PDLA)

**Scheme 1:**
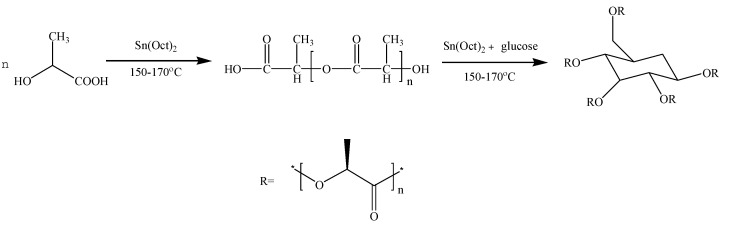
Synthesis route to PDLA and PDLAG.

The synthetic route of poly(d-lactic acid) is shown in Scheme 1 via direct melt polymerization [32]. A certain amount of d-LA was added into a three-necked flask and heated up to 150 °C under normal pressure for 1 h. Then, the catalyst stannous octoate with 0.5 wt% of the total mass of d-LA was added to the flask. The reactor was heated to 170 °C and reacted under a complete vacuum for 8 h. The obtained product was dissolved in chloroform and precipitated by adding methanol in excess. The separated precipitates were dried under vacuum at 50 °C for 10 h to obtain PDLA samples with a *M*_w_ of 19,000 and a PDI of 1.11, as determined by gel permeation chromatography (GPC).

#### 2.2.2. Preparation of Poly(d-lactic acid-*co*-glucose) Copolymer (PDLAG)

The synthetic route of PDLAG is shown in Scheme 1. After the obtained PDLA was melted, glucose with a mass fraction of 2 wt% of PDLA and stannous octoate with 0.5 wt% of the total mass of reactants were added to the flask. Then, the reaction proceeded at a pressure less than 1000 Pa for 6 h. The products were dissolved in chloroform and precipitation by excess methanol. The separated precipitates were dried under vacuum at 50 °C for 10 h to obtain PDLAG samples. The obtained PDLAG were multi-arm amphiphilic copolymers with the *M*_w_ of 15,600 and the PDI of 1.90 determined by GPC [32].

#### 2.2.3. Preparation and Modification of PLLA Film

Poly(l-lactic acid) was purified by the precipitation method and its *M*_w_ was 138,000 with its PDI being 1.54, as detected by GPC. Then, PLLA was dissolved in chloroform to prepare the solution with the mass concentration of 10 wt%. The PLLA solution was dropped into a polytetrafluoroethylene mold and dried at room temperature to prepare PLLA films with the thickness about 1 mm. Then, the PLLA film was cut into a 1 cm × 1 cm square with a flat surface for surface modification.

**Figure 1:**
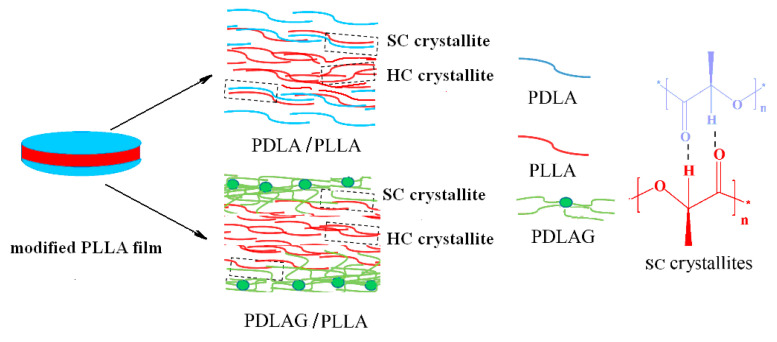
Structural schematic of modified PLLA film.

PDLA and PDLAG samples were dissolved in chloroform, respectively, to obtain the solutions with a mass concentration of 5 wt%. Then, PLLA films were immersed in PDLA or PDLAG solution for 0.5~3 min, then the upper surface and lower surface of the film were in contact with the solutions and the modification was performed homogeneously on both surfaces and the surrounding cross-sections. The optimal immersion time was determined by observing the state of PLLA films in the dissolutions. If immersion time was longer than 3 min, the swelling of PLLA film was clearly observed, and the PLLA film would become smaller due to partial dissolution or be broken into pieces. The modified PLLA films were washed with deionized water and dried under vacuum at 50 °C for 10 h. Here, m-PLLA stood for the surface-modified PLLA film in PDLA solution, while m-PLAG for the modified PLLA film in PDLAG solution. The structural schematic of modified PLLA films is shown in Figure 1. All modified PLLA samples are listed in Table 1.

### 2.3. Characterization Methods

An FT-IR-360 infrared spectrometer (Thermo Nicolet Corporation, Beijing, China) was used to measure the infrared spectra (FT-IR) of samples, with KBr tableting, scanning range being 500 to 4500 cm^−1^.

Gel permeation chromatography (GPC) was performed by Agilent 1100 gel permeation chromatography (Agilent Technologies (China) Co., Ltd., Shanghai, China) to measure the molecular weight of samples and tetrahydrofuran (THF) was used as the mobile phase to dissolve the sample in THF at a concentration of 1 mg/mL with a flow rate of 1 mL/min and an injection volume of 20 µL.

The thermal performance of the sample was measured using differential scanning (DSC) by means of the DSC-200F3 differential scanning calorimeter (NETZSCH-Gerätebau GmbH, Selb, Germany). The test temperature range was 10~250 °C. under a nitrogen gas flow of 30 mL/min, and the heating rate was 10 °C/min. The samples were first heated to 200 °C, kept for 3 min at this temperature and cooled rapidly to eliminate the heat history. The second heating process was recorded. Each sample was analyzed three times, and the DSC curve obtained each time were the same. The crystallinity was calculated by Proteus Analysis software.

Q5000 thermogravimetric analyzer (USA TA) was used to determine the TGA curve of the polymers with the temperature range from 20 to 600 °C at the heating rate of 10 °C/min, with the flow rate of N_2_ being 10 mL/min.

X-ray diffraction (XRD) analysis was performed with a Rigaku D/max-Ra X-ray diffractometer (Rigaku Corporation, Tokyo, Japan) with Cu-Kα radiation(λ = 0.154 nm), 40 kV working voltage, and electric current 30 mA. The range of 2θ was from 5° to 40° at a scan rate of 5°/min.

Polarized optical microscopy (POM) was used to observe the crystal morphology by means of the ECLIPSE polarizing microscope (Nikon Corporation, Tokyo, Japan). All samples were dried at 50 °C for 10 h before detection. 

The surface morphology of films was observed using the Quanta 200 scanning electron microscope (SEM) (USA FEI, Hillsboro, OR, USA).

The static water contact angles were characterized by the JC2000C1 contact angle meter (Shanghai Zhongchen Digital Technology Equipment Co., Ltd., Shanghai, China). The measurement time of each point was 10 s, and the contact angle was the average value of five measurements.

All polymer samples were prepared and measured at least three times, and the data from the repeated experiments had good reproducibility.

## 3. Results and Discussion

### 3.1. FT-IR Analysis of the Crystal Structure of Modified PLLA Films

**Figure 2:**
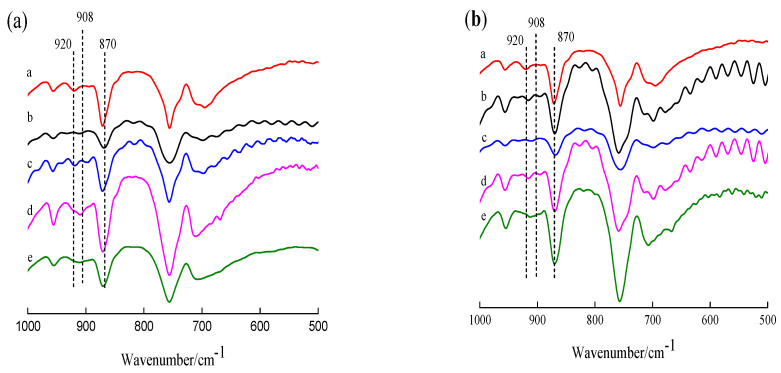
FTIR spectra of PLLA and sc-PLLA (**a**), and sc-PLAG (**b**). (**a**): a. PLLA; b. PDLA; c. m-PLLA-1; d. m-PLLA-2; e. m-PLLA-3; (**b**): a. PLLA; b. PDLAG; c. m-PLAG-1; d. m-PLAG-2; e. m-PLAG-3.

FT-IR was used to evaluate the changes in the crystalline structure of PLLA due to the surface treatments. All FT-IR spectra of PLLA and modified PLLA films were similar. In order to investigate the crystal structure of modified PLLA films, FTIR spectra only considered the crystalline regions. Figure 2 gave IR spectra of PLLA, PDLA, m-PLLA and m-PLAG in crystalline regions.

In Figure 2a, PLLA spectrum shows a peak at 870 cm^−1^ due to the PLLA amorphous phase and a peak at 920 cm^−1^ due to the homogeneous crystals (HC) of PLLA [33,34,35]. After modification using PDLA, the IR spectra of surface-modified PLLA showed a new peak at 908 cm^−1^ due to the stereocomplex crystals (SC) [33,36]. This result indicated that the surface of PLLA films was swelled in PDLA dissolution and then PLLA chains interacted with PDLA chains to form stereocomplex crystal structure on PLLA surface in a period of time. Meanwhile, the IR spectra of m-PLLA samples also had the peak at 920 cm^−1^ due to HC structure of PLLA in bulk as well as SC on the surface of m-PLLA. The intensity of the SC peak increased with the increase in surface modification time, and HC peak and the SC peak overlapped to eventually form one peak. The FT-IR spectra of modified PLLA film confirmed the formation of stereocomplex crystals on surface.

As show in Figure 2b, the IR spectra of m-PLAG samples were similar to those of m-PLLA samples, while the effect of modification time on the surface structure was alike, too. The FT-IR spectra of m-PLAG showed a peak at 908 cm^−1^ due to the SC structure as well as a peak at 920 cm^−1^ due to the HC structure. This result indicates that the SC structure formed on the PLLA surface owing to the interaction of PLLA chains with PDLA blocks of PDLAG, while the HC structure in the bulk of the polymer was affected due to the penetration of the polymer dissolutions into the bulk of the film. Moreover, the intensity of the SC peak of m-PLAG was weaker than that of m-PLLA with the same modification time, because the glucose residues in PDLAG chains may interact with PLLA and PDLA blocks, retard crystallization, and result in imperfect HC and SC structures [32,37].

The FT-IR results show that both PDLA and PDLAG could be used for the surface modification of PLLA films by stereocomplexation on the PLLA surface, while the HC structure of PLLA was retained in the bulk of PLLA films. Moreover, there was the interaction of glucose with lactic acid units.

### 3.2. Analysis of Thermal Performance of Modified PLLA Film

**Figure 3:**
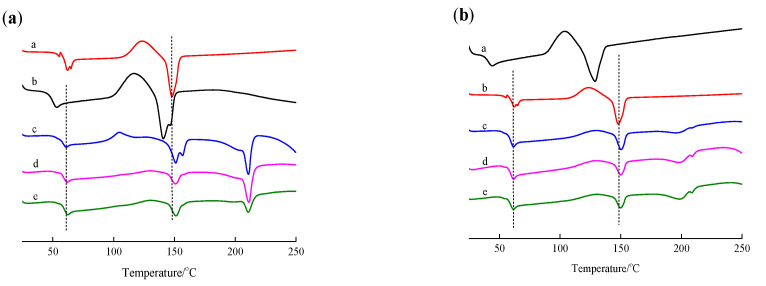
DSC curve of PLLA, PDLA, PDLAG, m-PLLA and m-PLAG samples. (**a**) a. PLLA; b. PDLA; c. m-PLLA-1; d. m-PLLA-2; e. m-PLLA-3; (**b**) a. PLLA; b. PDLAG; c. m-PLAG-1; d. m-PLAG-2; e. m-PLAG-3.

The thermal performance of PLLA films before and after surface modification was analyzed using the DSC method. Figure 3 shows the DSC curves of PLLA, PDLA, PDLAG, and PLLA films modified by PDLA and PDLAG, respectively. The glass transition temperature (*T*_g_), homogeneous crystal melting temperature (*T*_m,HC_) and stereocomplex crystal melting temperature (*T*_m,SC_) obtained from the DSC curve were shown in Table 2. The HC crystallinity (*f*_c,HC_) of all samples was calculated by the percentage of sample melting enthalpy and the melting enthalpy of PLLA with the crystallinity of 100% (93.6 J/g) [26,33]. The SC crystallinity (*f*_c,SC_) of the modified PLLA films was calculated by the percentage of sample melting enthalpy and the melting enthalpy of sc-PLA with 100% crystallinity (142 J/g) [38,39].

In Figure 3a, PLLA had the *T*_m_ of 147.9 °C due to the HC of l-lactic units [40] and the *T*_g_ of 61.9 °C, while PDLA had the *T*_m_ of 140.9 °C due to the HC formed by d-LA segment and the *T*_g_ of 52.8 °C. Compared with PDLA, PLLA with higher *M*_w_ possessed higher *T*_m_ and higher *T*_g_ [27]. In the DSC curves of m-PLLA films, the melting temperature due to SC structure (*T*_m,SC_) appeared at about 210 °C [3,41], while the melting temperature due to the HC structure of PLLA (*T*_m,HC_) remained at 150 °C, slightly higher than that of neat PLLA film. This result indicates that the stereocomplex crystal was formed on the surface of PLLA films during modification, while the HC structure in the bulk of the polymer was affected due to the swelling of the polymer during the surface treatment. The crystallinity of the PLLA film was 21.8%, while the crystallinity of the PDLA film was 18.1% owing to its low *M*_w_. The *f*_c,HC_ of m-PLLA was much lower than the crystallinities of PLLA and PDLA, and decreased gradually with increasing modification time. The *f*_c,SC_ of m-PLLA, as well as the sum of *f*_c,HC_ and *f*_c,SC_ of m-PLLA, decreased gradually with increasing modification time, while the sum of *f*_c,HC_ and *f*_c,SC_ was far less than the crystallinities of PLLA and PDLA. These results indicate that surface modification by immersing PLLA films in PDLA dissolution could destroy the imperfect HC structure of PLLA and form imperfect SC structure, which resulted in higher *T*_m,HC_ and lower *f*_c,HC_. As the modification time increases, the *T*_m,SC_ of m-PLLA was higher and the *f*_c,SC_ of m-PLLA was lower [42].

In Figure 3b and Table 2, PDLAG had a *T*_m,HC_ of 128.8 °C due to the HC of PDLA segments, a *T*_g_ of 44.2 °C and *f*_c,HC_ of 17.6%, which showed that PDLAG with lower *M*_w_ and glucose residues in chains may lead to crystal imperfection, so its *T*_m_, *T*_g_ and *f*_c,HC_ were lower than those of PDLA. In the DSC curves of m-PLAG films, the *T*_m,SC_ was 208 °C due to the SC of m-PLAG and the *T*_m,HC_ was 150 °C due to the HC of PLLA, which indicates that the SC structure appeared on the m-PLAG surface during modification, and the HC structure in the bulk of the polymer was affected due to the penetration of the polymer dissolutions into the bulk of the film. The *f*_c,HC_ of m-PLAG and the sum of *f*_c,HC_ and *f*_c,SC_ of m-PLAG were much lower than the crystallinities of PLLA and PDLAG, and decreased gradually with increasing modification time, while *f*_c,SC_ of m-PLAG increased slightly. These results indicate that surface modification by immersing PLLA films in PDLA and PDLAG dissolutions could destroy the imperfect HC structure of PLLA and form an imperfect SC structure, which results in higher *T*_m,HC_ and lower *f*_c,HC_ for both m-PLLA and m-PLAG compared with the neat PLLA. Compared with m-PLLA, m-PLAG had lower *T*_m,HC_, *T*_m,SC_ and *f*_c,SC_ [42]. The hydrophilic glucose groups interacting with PLLA and PDLA segments could cover the surface of PLLA films, and would prevent chloroform from approaching and dissolving PLLA films. Thus, the HC destruction of PLLA was retarded, and the *f*_c,HC_ of m-PLAG was slightly decreased with increasing immersion time compared with m-PDLA. The difference between m-PLAG and m-PLLA showed that the interaction of glucose residues with PLLA or PDLA segments, together with the hydrophilicity of glucose residues, could retard HC destruction and SC crystallization. Moreover, the PDI of PDLAG was less than that of PDLA, but its *M*_w_ was similar to that of PDLA. Therefore, PDLAG might possess more low *M*_w_ fractions, and low-*M*_w_ PDLAG would reduce SC crystallization and increase defects in the crystal structure. Thus, under the comprehensive effects of all of the above factors, m-PLAG possessed lower *T*_m,HC_, *T*_m,SC_ and *f*_c,SC_ than m-PLLA.

Table 3 presents one-way ANOVA for the *f*_c,SC_ of m-PLLA-1 and m-PLAG-1. The calculated *p*-value of 0.0002 was smaller than the test hypothesis α (0.05), which meant that PDLA and PDLAG had a significant effect on the *f*_c,SC_ of the modified PLLA films with a modification time of 0.5 min. When the modification time was 1 and 3 min, respectively, the results of the one-way ANOVA are similar to that with the 0.5 min modification time. The results of the one-way ANOVA for *f*_c,HC_ are similar to those of *f*_c,HC_. As for the one-way ANOVA for *T*_m,HC_ of m-PLLA and m-PLAG, the *p*-value (0.5551 and 0.8298) was larger than the test hypothesis α of 0.05 when the modification time was 0.5 and 1 min, which showed that PDLA and PDLAG had no significant effect on the *T*_m,HC_ of the modified PLLA films. When the modification time was 0.5 min, the *p*-value of 0.0936 was bigger than the test hypothesis α of 0.05, and showed PDLA and PDLAG had no significant effect on the *T*_g_ of the modified PLLA films. In order to compare the effects of PDLA and PDLAG on the modified PLLA, the one-way ANOVA was performed with PLLA with the test hypothesis α of 0.01. The *p*-value (near zero) was much smaller than α, indicating that PDLA and PDLAG had significant effects on the thermal properties of modified PLLA films.

The DSC results correspond with those of FT-IR. Both PDLA and PDLAG could be used for the surface modification of PLLA films by means of stereocomplexation on the surface of PLLA films, while the HC structure in the bulk of the polymer was affected due to the swelling of the polymer during the surface treatment. Moreover, the hydrophilicity of glucose residues and the interaction of glucose residues with lactic acid units could retard HC destruction and SC crystallization, which led to m-PLAG samples having lower *T*_m,HC_, *T*_m,SC_ and *f*_c,SC_.

**Figure 4:**
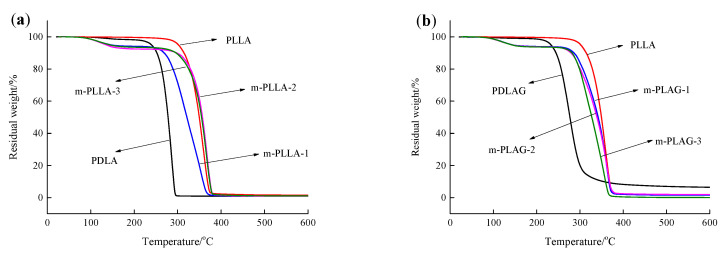
TGA curve of PLLA, PDLA, PDLAG, m-PLLA and m-PLAG samples. (**a**) PLLA, PDLA and m-PLLA; (**b**) PLLA, PDLAG and m-PLAG.

Thermogravimetric analysis (TGA) can also effectively evaluate the thermal properties of polymers. Figure 4 shows the TGA curves of PLLA, PDLA, PDLAG and modified PLLA films. The beginning degradation temperature (*T*_b_) (at 5 wt% mass loss), the maximum degradation temperature (*T*_max_) (at 50 wt% mass loss) and the residual carbon ratio (*W*_f_) at 600 °C obtained from the analysis of TGA curves are listed in Table 2. As seen in Figure 4 and Table 2, the *T*_b_ of PLLA film was higher than that of PDLA, PDLAG and modified PLLA films, which indicated that PLLA with high *M*_w_ was more stable at low temperature. The *T*_max_ of PLLA film was about 70 °C higher than that of PDLA and PDLAG and about 8~20 °C higher than that of m-PLAG, but it was slight lower than that of m-PLLA films. As modification time increased, the *T*_b_ and *T*_max_ of m-PLLA were higher than those of m-PLAG. These results show that the stereocomplex crystals formed on the surface of modified PLLA films, and there was still a HC structure in the bulk phase during surface modification. The crystal structure has important influence on the thermal properties of polymers. The SC structure could improve the heat resistance of PLA films. The rigidity of the glucose group and its strong interaction with PLA chains would confine the ordered arrangement of PLLA and PDLA chains and block crystallization so as to lessen the crystallinity of m-PLAG, which may promote the thermal degradation of PLA materials [43,44].

### 3.3. XRD Analysis of Modified PLLA Film

**Figure 5:**
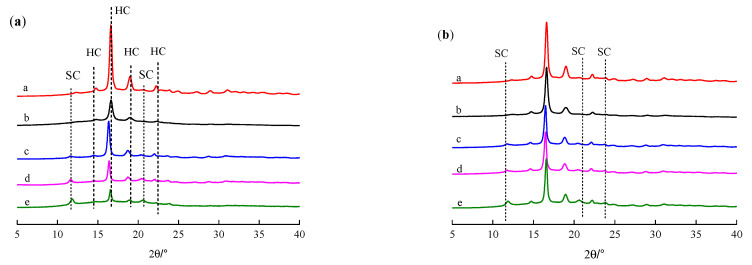
XRD curve of PLLA, PDLA, PDLAG, m-PLLA and m-PLAG samples. (**a**) a. PLLA; b. PDLA; c. m-PLLA-1; d. m-PLLA-2; e. m-PLLA-3; (**b**) a. PLLA; b. PDLAG; c. m-PLAG-1; d. m-PLAG-2; e. m-PLAG-3.

Figure 5 shows the XRD curves of the PLLA, PDLA, PDLAG and modified PLLA films, respectively. The XRD curves of PLLA, PDLA and PDLAG films show four diffraction peaks at 2θ of 14.7°, 16.6°, 18.9° and 22.3°, corresponding to the (010), (200)/(110), (203) and (015) planes of PLA α form crystal, respectively, i.e., PLLA, PDLA and PDLAG had a HC structure [45,46]. Moreover, the peak intensities of PDLA and PDLAG were obviously weaker than those of PLLA, indicating that low *M*_w_ and the addition of glucose could increase the crystal defects. All modified PLLA films presented diffraction peaks at 2θ of 11.9°, 20.7° and 23.9°, corresponding to the characteristic diffraction peaks of stereocomplex crystal, respectively [3,26,47], as well as weaker HC peaks. The intensity of the HC peaks of modified PLLA films decreased with increasing immersion time, while the intensity of SC peaks of modified PLLA films increased, which illustrated the trend of the SC structure on the surface and the HC trend in the bulk phase of modified PLLA. The XRD results are consistent with the above DSC conclusions.

### 3.4. POM Analysis of the Crystal Morphology of Modified PLLA Films

**Figure 6:**
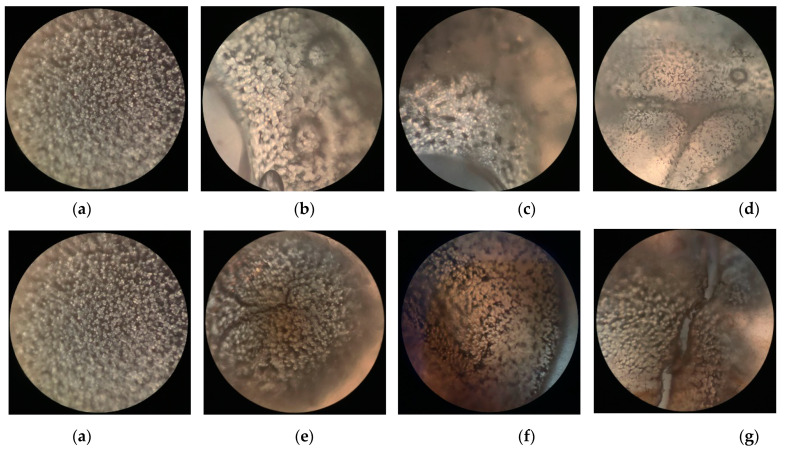
The polarizing microscope photographs of PLLA films before and after immersion in PDLA or PDLAG solution: (**a**) PLLA; (**b**) m-PLLA-1; (**c**) m-PLLA-2; (**d**) m-PLLA-3; (**e**) m-PLAG-1; (**f**) m-PLAG-2; (**g**) m-PLAG-3.

Figure 6 shows polarized optical microscope (POM) photographs of PLLA films before and after surface modification in PDLA or PDLAG dissolution. We can observe that the PLLA film had regular and uniform-sized spherulites with an obvious black cross extinction phenomenon [48,49]. As the modification time increased, the size and shape of the spherulites changed due to swelling and recrystallization, and the black cross extinction phenomenon disappeared. In addition, the crystal size of m-PLAG was smaller than that of m-PLLA with modification time accordingly, which implied that glucose residues could act as heterogeneous nucleation to promote crystallization and create more small crystallites [31,32].

### 3.5. SEM Analysis of the Morphology of Modified PLLA Films

**Figure 7:**
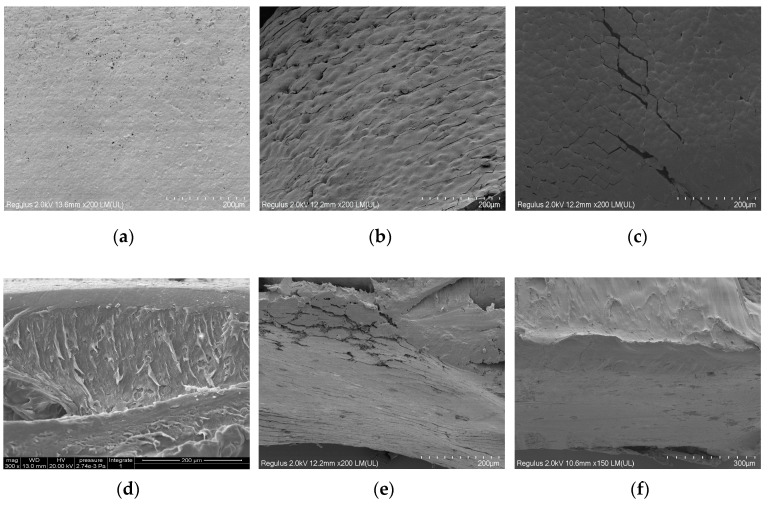
The SEM photographs o of PLLA and modified PLLA films: (**a**) surface of PLLA film; (**b**) surface of m-PLLA-3; (**c**) surface of m-PLAG-3; (**d**) cross section of PLLA film; (**e**) cross section of m-PLLA-3; (**f**) cross section of m-PLAG-3.

The SEM photographs of the surface and the cross section of PLLA film and its surface modified samples are given in Figure 7. The surface of PLLA film was uniform and the surface roughness was invisible.

Both m-PLLA-3 and m-PLAG-3 showed a damaged surface morphology after modification. In the cross-section of PLLA, there was a thin layer near to the surface which was morphologically different to the bulk of PLLA. The cross-sections of m-PLLA-3 and m-PLAG-3 were similar, which indicated that the swelling of the polymers could affect the entire volume of PLLA films as well as the film surface. These results show that the surface modification might affect the bulk structure of PLLA films, if the thickness of PLLA films were small and the modification time was long enough.

### 3.6. Hydrophilic Analysis of of Surface-Modified PLLA Films

**Figure 8:**
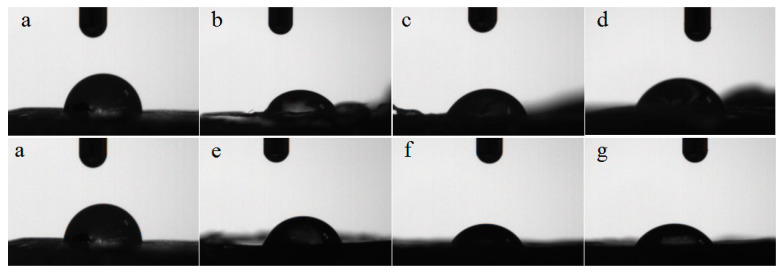
Water contact angle photographs of PLLA and modified films. (**a**) PLLA; (**b**) m-PLLA-1; (**c**) m-PLLA-2; (**d**) m-PLLA-3; (**e**) m-PLAG-1; (**f**) m-PLAG-2; (**g**) m-PLAG-3.

In order to study the hydrophilic properties of the PLLA films before and after modification, the water contact angle test of PLLA and its modified films was carried out. Figure 8 and Table 4 present the water contact angle pictures and parameters of the PLLA, m-PLLA and m-PLAG before and after modification, respectively.

We can see from Figure 8 and Table 4 that the contact angle of PLLA was 84.1°, indicating PLLA’s poor hydrophilicity. Compared with PLLA, the contact angles of m-PLLA and m-PLAG lessened, due to the damaged state and decreased crystallinity of the surface of modified PLLA films. The water contact angles of m-PLAG decreased significantly and were much less than those of m-PLLA, while the water contact angles of m-PLLA and m-PLAG all decreased with increasing immersion time. The m-PLAG-3 sample reached the smallest contact angle at 60.1°, which meant that it had the best hydrophilicity. All these outcomes suggest that the addition of hydrophilic glucose can significantly improve the hydrophilicity of PLA stereocomplex crystals [50].

## 4. Conclusions

The high-*M*_w_ PLLA was used to prepare PLLA films, and the PLLA films were surface-modified using low-*M*_w_ PDLA and PDLAG synthesized from d-LA and glucose through melt copolycondensation. Both PDLA and PDLAG could modify PLLA films through the stereocomplexation of enantiomeric PLA chains. The surface treatment destroyed most of the HC crystals of PLLA due to the swelling of the film, allowing the formation of some SC structures on the surface of the modified PLLA films. PLLA had a *T*_m_ of 147.9 °C and *f*_c,HC_ of 21.8%, while m-PLLA had a higher *T*_m,HC_ of 150.8 °C and a lower *f*_c,HC_ of 8.2%, as well as a *T*_m,SC_ of 210.8 °C and *f*_c,HC_ of 8.2%, along with m-PLAG having a *T*_m,HC_ of 150.6 °C and *f*_c,HC_ of 4.9%, as well as *T*_m,SC_ of 208.4 °C and *f*_c,HC_ of 0.4%. The m-PLLA films decreased both the HC and SC structure with increasing immersion time; the *f*_c,HC_ of m-PLLA varied from 8.2% to 3.1%, while the *f*_c,SC_ of m-PLLA varied from 8.2% to 2.1%. As the modification time increased, m-PLAG films increased the SC structure along with decreasing the HC structure, and the *f*_c,SC_ of m-PLAG varied from 0.4% to 1.2%, while the *f*_c,HC_ of m-PLAG varied from 4.9% to 4.1%, which was due to the effect of glucose residues.

Glucose residues of PDLAG sticking to the surface would improve the hydrophilicity of these films. Moreover, the hydrophilicity of glucose residues, the interaction of glucose residues with lactic acid units, and the effect of low-*M*_w_ PDLAG could retard HC destruction and SC crystallization and increase crystal defects, which led to m-PLAG samples having lower *T*_m,HC_, *T*_m,SC_, *f*_c,SC_ and water contact angles. The SC structure of modified PLLA film could improve its heat resistance, but glucose residues could block crystallization to promote the thermal degradation of PLA materials. The surface modification of PLLA films will improve the thermostability and the hydrophilicity of PLA materials and change the crystallization properties of PLA materials, which is essential in order to obtain PLA-based biomaterials. The surface-modified PLA films can be used in food packaging and biological materials. 

## Figures and Tables

**Table 1:** Modification time of PLLA films modified by PDLA and PDLAG.

Sample	Modification Time in PDLA Solution (min)	Sample	Modification Time in PDLAG Solution (min)
m-PLLA-1	0.5	m-PLAG-1	0.5
m-PLLA-2	1.0	m-PLAG-2	1.0
m-PLLA-3	3.0	m-PLAG-3	3.0

**Table 2:** DSC and TGA Thermal properties, crystallinity of polymer samples table.

Sample	Modification Time/min	*T*_m, HC_/°C	*T*_m, SC_/°C	*T*_g_/°C	*f*_c,HC_/%	*f*_c,SC_%	*T*_b_/°C	*T*_max_/°C	*W*_r_/%
PLLA	0	147.9	/	61.9	21.8	/	301.7	349.6	2.0
PDLA	0	140.6	/	52.8	18.1	/	239.7	278.1	0.9
PDLAG	0	128.8	/	44.2	17.6	/	233.1	276.8	6.5
m-PLLA-1	0.5	150.8	210.8	61.6	8.2	8.2	249.2	321.2	1.1
m-PLLA-2	1	151.0	211.2	62.4	3.4	5.7	278.1	357.1	1.4
m-PLLA-3	3	151.6	211.4	63.2	3.1	2.1	256.3	355.0	1.7
m-PLAG-1	0.5	150.6	208.4	61.2	4.9	0.4	253.7	342.1	2.0
m-PLAG-2	1	151.0	208.6	61.4	4.7	0.7	252.1	339.2	2.0
m-PLAG-3	3	150.2	208.8	61.8	4.1	1.2	249.3	327.4	0.6

**Table 3:** One-way ANOVA for *f*_c,SC_ of m-PLLA-1 and m-PLAG-1.

Sample	Number	Sum	Mean	Variance	/	/
m-PLLA-1	3	632.4	210.8	0.01	/	/
m-PLAG-1	3	625.2	208.4	0.09	/	/
Source of difference	SS	df	MS	F	*p*-value	F crit
Between groups	8.64	1	8.64	172.8	0.000193	7.708647
Within groups	0.2	4	0.05	/	/	/
sum	8.84	5	/	/	/	/

**Table 4:** Water contact angle parameters of PLLA and modified films.

Sample	Modification Time/min	Water Contact Angle/°
PLLA	0.0	84.1
m-PLLA-1	0.5	76.5
m-PLLA-2	1.0	72.5
m-PLLA-3	3.0	68.5
m-PLAG-1	0.5	69.5
m-PLAG-2	1.0	63.2
m-PLAG-3	3.0	60.1

## Data Availability

The data presented in this study are available on request from the corresponding author.
